# Entrapment Syndrome in a Kidney Transplant Recipient with Cryptococcal Meningitis

**DOI:** 10.3390/pathogens12050711

**Published:** 2023-05-13

**Authors:** Laya Reddy, George R. Thompson, Alan Koff, Stuart H. Cohen

**Affiliations:** 1 Department of Internal Medicine, Division of Infectious Diseases, University of California Davis Medical Center, Sacramento, CA 95817, USA; 2 Department of Medical Microbiology and Immunology, University of California—Davis, Davis, CA 95616, USA

**Keywords:** cryptococcosis, entrapment syndrome, cryptococcal meningitis, solid organ transplant

## Abstract

*Cryptococcus neoformans* primarily affects immunocompromised individuals and the central nervous system (CNS) is the most common site of dissemination. Entrapped temporal horn syndrome (ETH) remains a rare CNS manifestation and has not previously been described in solid organ transplant recipients. Here, we present a case of ETH in a 55-year-old woman with history of renal transplant and prior treated Cryptococcal meningitis.

## 1. Introduction

*Cryptococcus neoformans* (*C. neoformans)* is an encapsulated yeast pathogenic to both humans and animals [1,2]. *C. neoformans* has been found in soil worldwide and in the avian environment in particular [2]. As an opportunistic pathogen, *C. neoformans* primarily affects those who are immunocompromised due to conditions such as acquired immunodeficiency syndrome, organ transplantation on immunosuppressive agents, or malignancy on anti-cancer drugs [1,3]. Specifically, among solid organ transplant recipients who contracted invasive fungal infections, cryptococcal infection occurred in approximately 8% of the patients, with most cases occurring after 12 months following transplantation [4,5].

Infection occurs primarily through inhalation of the fungus. Due to a thick polysaccharide capsule, *Cryptococcus* survives within pulmonary macrophages [1]. *C. neoformans* can migrate from the lungs to the bloodstream or meninges if not contained by the patient’s immune system [1]. The central nervous system (CNS) is the most common site of disseminated infection; however, the skin and soft tissue, eyes, bones, prostate, and lymph nodes may all be involved as well. In kidney transplant patients with cryptococcal meningitis, diagnostic delays occur due to non-specific symptoms, and the mortality rate can be as high as 40% at 12 months [5,6].

Complications of CNS infection include meningoencephalitis, cranial neuropathies, brain abscess, vasculitis, ischemic or hemorrhagic stroke, and communicating or non-communicating hydrocephalus. Temporal horn entrapment represents a rare form of isolated non-communicating hydrocephalus and has not previously been described in association with cryptococcosis in solid organ transplant patients. Here, we report a case of an immunocompromised kidney transplant recipient who developed temporal horn entrapment following treatment of *C. neoformans* CNS infection.

## 2. Case Presentation

This case describes a 55-year-old woman with a history of recurrent kidney stones, which led to kidney failure. She was maintained on hemodialysis for 6 years prior to kidney transplantation. She received thymoglobulin induction therapy at the time of transplantation. She was maintained on tacrolimus 1.5 mg once every morning and 1 mg once every evening, mycophenolate mofetil 750 mg twice daily, and prednisone 5 mg daily. Post-transplant course initially presented with delayed graft function, but she eventually was able to discontinue dialysis and currently has good allograft function, with a baseline creatinine of 1.0 mg/dL. She had no history of rejection.

Her cryptococcal history was pertinent for headaches beginning 5 months after renal transplantation. She was evaluated in the emergency department multiple times and was initially diagnosed with migraine headaches. At 11 months post-transplant, her headaches worsened, and she developed nausea and vomiting, prompting a lumbar puncture to be performed, which showed an opening pressure of 24 cm H_2_O, elevated cerebrospinal fluid (CSF), elevated white blood cells (115 per mm^3^), minimally elevated CSF red blood cells (10 per mm^3^), low CSF glucose (4 mg/dL), elevated CSF protein (164 mg/dL), positive Cryptococcal CSF antigen, and a yeast which, on blood and chocolate agar, was identified as *C. neoformans* using Matrix-Assisted Laser Desorption/Ionization (MALDI). She received induction therapy with liposomal amphotericin B 5 mg/kg IV daily and flucytosine 1500 mg PO QID. After 2 weeks of treatment, opening pressure normalized, CSF white blood cells dropped to 53 per mm^3^, CSF glucose rose to 18 mg/dL, and protein dropped to 132 mg/dL; Cryptococcal CSF antigen was negative, and repeat CSF cultures were negative. Following 2 weeks of induction therapy, she was transitioned to fluconazole 600 mg PO daily for 3 months, followed by fluconazole 200 mg PO daily thereafter, based on her renal function. She reported excellent adherence.

**Figure 1:**
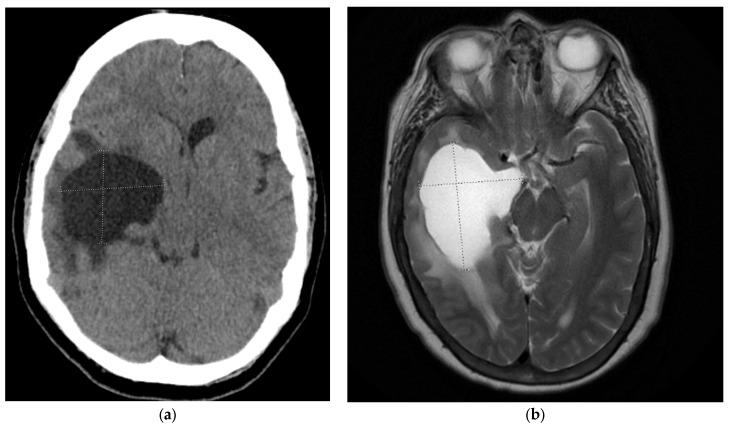
(**a**,**b**) Computed tomography (CT) head and T2-weighted magnetic resonance imaging (MRI) demonstrates a circumscribed cystic structure/lesion effacing the right temporal lobe with surrounding vasogenic edema and midline shift consistent with entrapment syndrome.

At 21 months post-transplant (10 months after initiation of cryptococcal therapy), she developed worsening headaches, nausea, and vomiting. *Computed tomography (CT)* head without contrast revealed a 4.9 × 4.3 × 4.8 cm circumscribed cystic structure/lesion effacing the right temporal lobe with surrounding vasogenic edema and midline shift (Figure 1a). Magnetic resonance imaging revealed similar findings (Figure 1b). She was started on liposomal amphotericin B 5 mg/kg IV, flucytosine 1500 mg PO QID, and dexamethasone. The patient’s tacrolimus and prednisone were continued, and mycophenolate mofetil was held. She underwent ventriculoperitoneal (VP) shunt placement for decompression. A lumbar puncture at that time revealed a CSF white blood cell count (8 per mm^3^), elevated red blood cell count (23 per mm^3^), mildly elevated protein (47 mg/dL), high glucose (73 mg/dL), negative meningoencephalitis panel (BioFire Diagnostics LLC, Salt Lake City, UT, USA), negative bacterial/fungal cultures, and negative CSF cryptococcal antigen testing. Following the return of CSF results, liposomal amphotericin B was discontinued and home medications of mycophenolate mofetil, fluconazole, and prednisone were restarted. Her presentation of a new trapped right temporal horn was thought to be a complication of her prior episode with *C. neoformans* meningitis due to intraventricular scarring. Her course was complicated by occipital neuralgia related to the VP shunt and was managed with gabapentin. She completed a total of 26 months of total therapy, after which fluconazole was stopped. At 9 months after the cessation of therapy, she is doing clinically well without a return of prior cryptococcal meningitis.

## 3. Discussion

Entrapped temporal horn syndrome (ETH) is a form of focal hydrocephalus in which the obstruction of one lateral ventricle in the region of the trigone isolates the temporal horn. Due to the continued secretion of cerebrospinal fluid within the temporal horn, the pressure increases with a surrounding mass effect, mimicking a mass lesion. Following focal ventriculitis within the temporal horn and the accompanying elevated CSF protein concentration, meningeal inflammation and the increased CSF viscosity may cause obstruction of the foramen of Monro. This obstruction impairs normal CSF flow from the lateral ventricles to the third ventricle [7].

Infectious causes of a trapped temporal horn, such as meningitis, ventriculitis, and hydatid cysts, as well as intraventricular neurocysticercosis and the development of fibrosis, are thought to be predisposing factors [8,9,10]. Meningitis and ventriculitis are thought to damage the ventricular wall ependyma, causing glial tissue tuft growth and subsequent septation, which can continue to enlarge and advance even with control of the underlying meningitis [9,10,11]. Clinical manifestations include headache, visual field defects, and even seizures and hemiparesis [10].

Previously, there have been reports in the literature involving mass lesions caused by choroid plexus involvement due to cryptococcal infection. One case in an immunocompetent patient demonstrated that choroid inflammation from cryptococcal meningitis can lead to the formation of intraventricular synechiae, loculation, and temporal horn entrapment [12,13]. Findings of cryptococcomas or a gelatinous pseudocyst in the choroid plexus were specific for CNS cryptococcosis, but unilateral or bilateral enlargement of the choroid plexus is rare [7,14].

Because the choroid plexus is the interface between the CSF and systemic circulation, it is the portal of entry for many pathogens, including cytomegalovirus, tuberculosis, and other infectious agents, in addition to *Cryptococcus* spp. Zhuang et al. previously described a case of presumed bacterial meningitis complicated by an entrapped temporal horn two months after successful antimicrobial treatment [10]. Another case described a young woman with central nervous system tuberculosis which led to a trapped right temporal horn and focal hydrocephalus [14].

The treatment of entrapment syndrome consists of surgical intervention to alleviate the obstruction, and this can be performed endoscopically. Given that this complication may occur in the absence of ongoing presence of pathogens, fluid analysis and cultures for the assessment of disease quiescence is important to guide ongoing therapy. Surgical treatment is paramount and includes re-establishing communication of the trapped ventricle with the rest of the ventricular system, or providing a shunt system, as in our patient [12].

In summary, ETH should be suspected when patients develop symptoms of an expanding temporal lobe mass following a condition involving the trigone of the lateral ventricle. The diagnosis should also be considered in patients who have a history of cryptococcal meningitis with imaging findings of a large cystic structure centered within the temporal lobe.

## Data Availability

Not applicable.

## References

[1] Chen Y., Shi Z.W., Strickland A.B., Shi M. (2022). *Cryptococcus neoformans* Infection in the Central Nervous System: The Battle between Host and Pathogen. J. Fungi.

[2] Rathore S.S., Sathiyamoorthy J., Lalitha C., Ramakrishnan J. (2022). A holistic review on Cryptococcus neoformans. Microb. Pathog..

[3] Jackson K.M., Ding M., Nielsen K. (2023). Importance of Clinical Isolates in *Cryptococcus neoformans* Research. J. Fungi.

[4] Pappas P.G., Alexander B.D., Andes D.R., Hadley S., Kauffman C.A., Freifeld A., Anaissie E.J., Brumble L.M., Herwaldt L., Ito J. (2010). Invasive fungal infections among organ transplant recipients: Results of the Transplant-Associated Infection Surveillance Network (TRANSNET). Clin. Infect. Dis..

[5] Tardieu L., Divard G., Lortholary O., Scemla A., Rondeau É., Accoceberry I., Agbonon R., Alanio A., Angoulvant A., Albano L. (2022). Cryptococcal Meningitis in Kidney Transplant Recipients: A Two-Decade Cohort Study in France. Pathogens.

[6] Van der Torre M.H., Andrews R.A., Hooker E.L., Rankin A., Dodd S. (2022). Systematic review on *Cryptococcus neoformans*/*Cryptococcus gattii* species complex infections with recommendations for practice in health and care settings. Clin. Infect. Pract..

[7] Sharma C., Acharya M., Kumawat B.L., Kochar A. (2014). ‘Trapped temporal horn’ of lateral ventricle in tuberculous meningitis. BMJ Case Rep..

[8] Maurice-Williams R.S., Choksey M. (1986). Entrapment of the temporal horn: A form of focal obstructive hydrocephalus. J. Neurol. Neurosurg. Psychiatry.

[9] Shaariah W., Morad Z., Suleiman A.B. (1992). Cryptococcosis in renal transplant recipients. Transplant. Proc..

[10] Zhuang Y., Richard S., Zhou J., Liu J., Fang Z., Chen Z. (2022). Entrapped temporal horn syndrome: A retrospective analysis of 5 case series. Int. J. Surg. Glob. Health.

[11] Rhoton A.L., Gomez M.R. (1972). Conversion of multilocular hydrocephalus to unilocular. Case report. J. Neurosurg..

[12] Krähenbühl A.K., Baldauf J., Gaab M.R., Schroeder H.W. (2013). Endoscopic temporal ventriculocisternostomy: An option for the treatment of trapped temporal horns. J. Neurosurg. Pediatr..

[13] Kovoor J.M., Mahadevan A., Narayan J.P., Govindappa S.S., Satishchandra P., Taly A.V., Shankar S.K. (2002). Cryptococcal choroid plexitis as a mass lesion: MR imaging and histopathologic correlation. AJNR Am. J. Neuroradiol..

[14] Kumari R., Raval M., Dhun A. (2010). Cryptococcal choroid plexitis: Rare imaging findings of central nervous system cryptococcal infection in an immunocompetent individual. Br. J. Radiol..

